# Re-evaluating the diagnostic efficacy of PSA as a referral test to detect clinically significant prostate cancer in contemporary MRI-based image-guided biopsy pathways

**DOI:** 10.1177/20514158211059057

**Published:** 2021-12-01

**Authors:** Artitaya Lophatananon, Alexander Light, Nicholas Burns-Cox, Angus Maccormick, Joseph John, Vanessa Otti, John McGrath, Pete Archer, Jonathan Anning, Stuart McCracken, Toby Page, Ken Muir, Vincent J Gnanapragasam

**Affiliations:** 1 Division of Population Health, Health Services Research & Primary Care Centre, University of Manchester, UK; 2 Division of Urology, Department of Surgery, University of Cambridge, UK; 3 Department of Urology, Cambridge University Hospitals NHS Foundation Trust, UK; 4 Department of Urology, Somerset NHS Foundation Trust, UK; 5 Department of Urology, Royal Devon and Exeter NHS Foundation Trust and University of Exeter, UK; 6 Department of Urology, Southend Hospital, UK; 7 Department of Urology, North Bristol NHS Trust, UK; 8 Department of Urology, South Tyneside and Sunderland NHS Trust, UK; 9 Department of Urology, Newcastle Hospitals NHS Trust, UK; 10 Cambridge Urology Translational Research and Clinical Trials Office, Addenbrooke’s Hospital, UK

**Keywords:** Prostate cancer, PSA, PSA density, MRI, diagnosis, predictive value

## Abstract

**Introduction:**

Modern image-guided biopsy pathways at diagnostic centres have greatly refined the investigations of men referred with suspected prostate cancer. However, the referral criteria from primary care are still based on historical prostate-specific antigen (PSA) cut-offs and age-referenced thresholds. Here, we tested whether better contemporary pathways and biopsy methods had improved the predictive utility value of PSA referral thresholds.

**Methods:**

PSA referral thresholds, age-referenced ranges and PSA density (PSAd) were assessed for positive predictive value (PPV) in detection of clinically significant prostate cancer (csPCa – histological ⩾ Grade Group 2). Data were analysed from men referred to three diagnostics centres who used multi-parametric magnetic resonance imaging (mpMRI)-guided prostate biopsies for disease characterisation. Findings were validated in a separate multicentre cohort. Results: Data from 2767 men were included in this study. The median age, PSA and PSAd were 66.4 years, 7.3 ng/mL and 0.1 ng/mL^2^, respectively. Biopsy detected csPCa was found in 38.7%. The overall area under the curve (AUC) for PSA was 0.68 which is similar to historical performance. A PSA threshold of ⩾ 3 ng/mL had a PPV of 40.3%, but this was age dependent (PPV: 24.8%, 32.7% and 56.8% in men 50–59 years, 60–69 years and ⩾ 70 years, respectively). Different PSA cut-offs and age-reference ranges failed to demonstrate better performance. PSAd demonstrated improved AUC (0.78 vs 0.68, *p* < 0.0001) and improved PPV compared to PSA. A PSAd of ⩾ 0.10 had a PPV of 48.2% and similar negative predictive value (NPV) to PSA ⩾ 3 ng/mL and out-performed PSA age-reference ranges. This improved performance was recapitulated in a separate multi-centre cohort (*n* = 541).

**Conclusion:**

The introduction of MRI-based image-guided biopsy pathways does not appear to have altered PSA diagnostic test characteristics to positively detect csPCa. We find no added value to PSA age-referenced ranges, while PSAd offers better PPV and the potential for a single clinically useful threshold (⩾0.10) for all age groups.

**Level of evidence:**

IV

## Introduction

Prostate cancer is the most common male cancer worldwide and the number of men who will need investigation is growing.^1^ Prostate multi-parametric magnetic resonance imaging (mpMRI) has revolutionised the diagnostic paradigm particularly in improving biopsy accuracy and hence better disease burden characterisation. Current best practice is therefore a pre-biopsy MRI to both establish if a lesion is present and to guide biopsy taking.^2–4^ While it is well established that imaging-based pathways can reduce unnecessary biopsies, it is less clear about how it has altered the disease spectrum seen at diagnosis.^5–8^ This is particularly pertinent as the entry point to the diagnostic pathway has remained unchanged, that is, prostate-specific antigen (PSA) tests done in the community as a positive reflex test and referral if it exceeds a pre-specified threshold. These thresholds and their diagnostic performance were based on decades old historical and out-dated random sampling biopsies of the prostate.^9,10^ It is now known that many men were likely missed or misclassified using these older method.^11,12^ This undoubtedly contributed to the modest sensitivity and specificity for PSA in finding cancers. In many countries, PSA age-reference standards are also recommended.^13–15^ These reference ranges are similarly based on historical cohorts and older models of biopsy practice.^16–19^ The diagnostic performance of historical PSA thresholds and PSA age-reference ranges has not since been re-evaluated in the modern image-based biopsy pathway. In particular, whether they perform better or worse in the context of image-based case selection and more accurate targeted biopsies.

In this retrospective multicentre cohort study, we addressed this question and re-assessed the detection value of current PSA cut-offs and age-reference PSA referral thresholds when calibrated against image-based pathways and biopsies for the detection of clinically significant prostate cancer (csPCa). We hypothesised that more accurate case selection and biopsy methods might have improved the performance of PSA as a positive discriminatory test or may suggest new optimal thresholds.

## Methods

### Cohorts

#### Primary cohort

Retrospective data from men referred between 2013 and 2020 to three geographically distinct UK prostate cancer secondary care diagnostics centres were used for this analysis. De-identified data were collected under individual institutional approvals as research or clinical audit (Cambridge University Hospitals R&D department REC 03/018, Devon and Exeter R&D department audit number 15-2058, Taunton and Somerset R&D department audit number 0236). As only fully anonymised data were used for this study, individual informed consent was not deemed necessary under the institutional approvals. All were referred from primary care for elevated PSA or abnormal digital rectal examinations (DRE). Men underwent pre-biopsy prostate MRI according to local protocols and reporting using the LIKERTS (Cambridge) or PIRADS V1 and more latterly V2 (Devon and Taunton) scoring system. MRI on 1.5T or 3T systems was performed, including standard T2, diffusion-weighted and contrast-enhanced sequences. Image acquisition and processing was performed in accordance with local standard clinical protocols as was the method and extent of biopsies. As our focus was on men who did proceed to biopsy, those who did not were not included in this study. No central reporting or standardisation was used thus our study cohort represents real-world practice. The estimated prostate volume from MRI was calculated using the ellipsoid formula. Exclusion criteria included men with a previous biopsy, pelvic metalwork interfering with MRI quality. Following MRI, men underwent image-guided targeted and systematic biopsies, either by cognitive or image fusion based on the centres practice. Men with negative MRIs, but ongoing suspicion had systematic biopsies only.

#### Validation cohort

Data collected as part of the previously reported PRIM (Phi to tRIage Mri) study were used to retest the findings and have been previously described.^20^ Briefly, the PRIM study was a five-centre prospective collection of data and serum for biomarkers to refine the use of MRI. Like the primary cohort, data were collected on age, PSA, prostate volume, PSA density (PSAd) and the detection of csPCa from a combination of image-based targeted and systematic biopsies. Only one centre was common to both internal and external cohorts, and there was no overlap in cases used in the analysis.

### PSA thresholds and reference ranges

PSA at diagnosis (pre-biopsy) was available in all men and PSAd calculated using MRI-defined prostate volumes (PSA divided by prostate volume). PSA cut-offs were based on the National Institute for Health and Care Excellence (NICE) and Public Health England guidance to general practitioners (GPs; https://cks.nice.org.uk/topics/prostate-cancer/diagnosis/psa-testing/ and https://www.gov.uk/government/publications/prostate-specific-antigen-testing-explanation-and-implementation) and the UK Prostate Cancer Risk Management Programme guidance (https://www.gov.uk/government/publications/prostate-cancer-risk-management-programme-psa-test-benefits-and-risks/prostate-cancer-risk-management-programme-pcrmp-ben-efits-and-risks-of-psa-testing#the-psa-test). Both documents refer only to a single PSA threshold at ⩾ 3 ng/mL for men aged 50–69 years, but no reference threshold for other ages. For men aged ⩾ 70 years, we used the most common guidance from Cancer Alliances, that is, PSA of ⩾ 5ng/mL.^21^

### Outcomes and statistical analysis

Our primary goal was to test whether PSA performance in regard to detecting csPCa (positive detection) has improved with modern MRI-based image-guided pathways. The definition of csPCa was based on International Society of Urological Pathology (ISUP) Grade Group 2 disease or higher (⩾ GG2) in those men who proceed to full investigations, that is, both mpMRI and image-guided biopsy. MRI lesion presence, absence, and scoring and association with diagnostic yields were not focuses of this study. Patient characteristics were summarised using descriptive statistics. Median and range were given for normally distributed continuous variables. Sensitivity, specificity, negative predictive value (NPV) and positive predictive value (PPV) were calculated for each test using *diagti* command.^22^ Prostate volume means were compared using Student’s t-test. We first assessed the use of different referral thresholds of PSA across all ages and stratified by age ranges in diagnosing csPCa. This was similarly done for PSAd with different referral thresholds. For PSA age reference, we tested two models: Model 1 – ⩽ 50 years: PSA ⩾ 2.5, 50–69 years: PSA ⩾ 3 ng/mL and 70–79 years: PSA ⩾ 5 ng/mL. Model 2 is a combination of PSA ⩾ 2.5 for men under 50 years or PSA ⩾ 3 everyone else. The proportions of men with positive test results were used to calculate diagnostic test characteristics and especially PPV. In the external PRIM cohort, we further tested the performance of PSA and PSAd against the additional outcomes of significant cancers defined as Cambridge Prognostic Group 2 (⩾CPG2) and Cambridge Prognostic Group 3 (⩾CPG3).^23^ These CPG groups are similar to the AUA classifications of favourable (CPG2) and unfavourable intermediate-risk disease (CPG3), respectively. All analysis was performed using the Stata statistical package, release 15 (StataCorp., College Station, TX, USA).

## Results

### Primary cohort characteristics

The primary study cohorts comprised 2767 men and are detailed in Table 1. The median age was 66.4 years (range, 36–90 years), median PSA 7.27 (0.1–4751 ng/mL) and median PSAd 0.11 (0.003–148 ng/mL^2^; Table 1). Any cancer type was detected in 61.1% of the cohort and csPCa (⩾GG2) in 38.7%.

### PSA threshold and age-referenced performance characteristics

PSA had an overall area under the curve (AUC) of 0.61 for detection of any cancer and 0.68 for csPCa (⩾GG2) which is similar to previously reported performance characteristics in the pre-MRI era (AUC = 0.64–0.70).^24–28^ Based on a single PSA threshold of ⩾ 3 ng/mL, the overall PPV was 40.3% for csPCa although performance characteristics did vary by age group (PPV in 50–59 years, 24.8%, 60–69years, 32.7% and ⩾ 70 years, 56.8%; Table 2). Conversely, NPV was better in younger men. Testing at different PSA cutoffs only showed marginally better PPV, but at significantly poorer NPV (Table 2). To test age-stratified PSA ranges, we used two models as described (Table 3). Model 1 produced an overall PPV of 40.2% and NPV of 83.8% for csPCa. An alternate model (Model 2; retaining the ⩽ 50 years: PSA ⩾ 2.5, but applying the PSA ⩾ 3 ng/mL cut-off for all other ages) had identical performance characteristics. Overall, both models performed very similarly to a single PSA ⩾ 3 ng/mL threshold in predicting csPCa suggesting no specific benefit of age-reference ranges in improving diagnostic test characteristics.

### Association of prostate volume with age

Given these findings, we re-challenged the base assumption underpinning PSA age-reference ranges, that is, that PSA rises with longevity related to increasing prostate volumes. To do this, we used objectively measured MRI-derived gland volumes across different age groups. Median prostate volumes did increase with age: 30 mL in men ⩽49years, 45.0 mL in men 50–59 years, 52.7 mL in men 60–69 years and 55.0 mL in those with 70–79 years (Table 4). Between the first three age groups, there was a significant incremental difference in size (*p* < 0.008). However, prostate volumes were not significantly different between men aged 60–69 years and 70–79 years (*p* = 0.18; Table 4). We further observed large variability in the range of sizes within any given age group suggesting that while prostate volumes may trend to increase with age, this is not a linear or inevitable association. Hence, pre-defined age-related thresholds are unlikely to perform well which is indeed what we had observed from the data above. Finally, we tested for any relationship between prostate volume itself and cancer detection. Here, we found that mean gland volume was higher in men with a benign diagnosis (68.1 mL, *SD* = 35.5) compared to any cancer or significant cancer diagnosis (52.5 mL, *SD* = 29.0 and 51.9 mL, *SD* = 30.0, respectively; *p* < 0.0001 for both).

### Utility of PSAd

Variability in prostate volume suggest that correcting individual PSA for gland size (PSAd) may be better for improving detection of csPCa. Indeed, the overall AUC for PSAd was significantly better compared to PSA alone for both any cancer (0.71 vs 0.61) and csPCa (0.78 vs 0.69; both *p* < 0.0001; Table 5) and again mirrored older pre-MRI studies on better PSAd performance as a diagnostic test.^25–27^ In terms of PPV, PSAd did improve upon PSA especially at higher PSAd thresholds (PSAd ⩾ 0.10 and ⩾ 0.15) with less of an effect in poorer NPV (Table 6). PSAd ⩾ 0.10 in particular had an improved PPV of 48.2% and NPV of 85.5%, while PSA ⩾ 3 ng/mL had a poorer PPV of 40.3%, but similar NPV (84.0%; Tables 2 and 6). PSAd ⩾ 0.15 had an even higher PPV, but at the expense of a worse NPV than PSA ⩾ 3 ng/mL. Although PSAd test characteristics also varied by age, they generally performed better in terms of PPV and NPV compared to PSA within each age group. A single PSAd threshold of ⩾ 0.10 also out-performed both age-reference Models 1 and 2 in term of PPV while retaining similar NPV (Table 3).

### External validation of test performance

To validate the above results, we retested the findings in the PRIM study cohort.^20^ Complete data from 541 men were available for this analysis and cohort characteristic are shown in Supplementary Table S1. Here, the overall PPV for detection of csPCa (defined as ⩾ GG2) for Models 1 and 2 was 48.9% for both, while PSAd ⩾ 0.10 showed an improved PPV of 54.4% (Supplementary Table S2). Better performance for PSAd ⩾ 0.10 remained true with different definitions of csPCa using the CGP system.^23^ For detection of ⩾ CPG2 and ⩾ CPG3, PSAd ⩾ 0.10 had a PPV of 61.1% and 44.2% compared to 54.6% and 38.3%, respectively for PSA age-reference models. NPV was also better with PSAd regardless of definition used (Supplementary Table S2).

## Discussion

In this study, we re-explored the utility of PSA thresholds and PSA age-reference ranges to predict the presence of csPCa in the context of UK real-world modern image-guided patient selection and biopsy practice. We find that PSA performance characteristics have not in fact improved compared to historical series, thus disproving our hypothesis. PSAd instead may offer better PPV while preserving NPV. A single PSAd threshold (PSAd ⩾ 0.10) also had better performance characteristics than PSA at any single cutoff or age-reference range.

The impact of image-based biopsy on the types of cancers detected in contemporary practice remains debated with the only consistent finding being reduced insignificant cancers. In at least four randomised-controlled trials, the positive detection rates of csPCa between non-image-based and MRI-informed biopsy approaches were not significantly different.^5–8^ In a very recent paper, Eklund et al.^29^ found that in a screening context using PSA ⩾ 3 ng/ mL and MRI-based biopsies found similar rates of csPCa compared to conventional investigations (21% vs 18%), and the main benefit was reduced rates of insignificant cancers detected. In contrast, two systemic review (including large numbers from single centre cohort studies) have suggested superiority of image-based approaches.^30,31^ Other studies comparing before and after introduction of MRI have also not found strong evidence of an increase in csPCa detection.^32^ These studies were based on differing PSA guidelines and thresholds depending on the population investigated. The definition of csPCa also varies between studies. Thus, it is as yet unclear if new biopsy methods have altered the diagnostic efficacy of the PSA test itself.

PSA is an invaluable first biomarker in prostate diagnostics and it is hard to envision pathways that do not incorporate it in some way.^33,34^ In the seminal work by Oesterling et al.^16^ undertaken over 30 years ago, PSA levels were correlated with age. This study and others informed the basis for PSA age-reference ranges which has remained largely unchanged since then.^16–19^ There is also little standardisation between and within countries. Across England, for instance, we recently reported at least 10 different PSA age-reference ranges being used which affected both referral likelihood and diagnosis of csPCa.^21^ In the current study, despite more accurate biopsy methods, the diagnostic performance of PSA reference ranges did not add value to single PSA cut-offs. We find that this is likely due to a very variable relationship between age and prostate volume and by association, different levels of endogenous PSA expression. There is also previous evidence that gland volume may have an inverse correlation with prostate cancer incidence though these studies were done in the pre-MRI era.^35,36^ Interestingly, in this study using MRI-based diagnostics, we were able to recapitulate this observation with benign cases having overall larger volumes compared to glands with cancer detected. The mechanisms of this are unclear though some have proposed that it may be due to a mass compressive effect.^37^ This observation does need further validation in future studies which also include men who do not proceed to biopsy after a negative MRI.

New biomarkers (mainly based on PSA and its derivatives) have shown better detection characteristics compared to PSA alone.^38,39^ A common theme of these markers is refinements that account for both the benign and malignant prostate secretory components.^40,41^ PSAd is the simplest example of this and has been consistently shown to be a better marker for prostate cancer presence compared to PSA.^25–27^ It has also been shown to have important clinical utility as a predictor of disease aggressiveness, progression in active surveillance, post-treatment failure and is an important inclusion in multi-component risk-calculators.^42–45^ Its popularity has in fact grown, since the advent of MRI as it is an important adjunct tool when a scan is negative.^46,47^ In this study, we recapitulated previous findings that show that PSAd is superior to PSA and also demonstrate that a single PSAd cut-off can be reasonably reliably applied across different age groups with improved PPV and similar NPV. Although not perfect, it appears a better first test for referral and MRI can add further incremental diagnostic sensitivity and specificity. Of note, we identified PSAd ⩾ 0.10 as the optimal cut-off, whereas it is PSAd ⩾ 0.12 or 0.15 which is recommended if a MRI scan is negative.^46–48^ This suggests that different PSAd cut-offs may be more relevant depending on the application context, indeed, in this study, PSAd ⩾ 0.15 had higher PPV, but poorer NPV compared to standard age-referenced models.

The obvious question is how can prostate volumes to generate PSAd be measured as a community-based test? PSAd has never gained traction likely because of this logistical issue. However, in the modern era, a number of developments may be making PSAd an increasingly plausible primary reflex test: (1) MRI, which is now mandated for nearly all men with suspected prostate cancer, is a resource-intensive tool with variable reporting heterogeneity. As such, refining its use has been identified as a research priority.^48–50^ Work in our unit has previously shown that up to 40% of men do not go onto a biopsy after an MRI, which is a significant waste of resources.^51^ (2) High-quality handheld transrectal ultrasound devices with automated prostate volume measurements are now available at a fraction of the cost of traditional machines. These are no more invasive than a DRE and could be incorporated into GP practices or community-based diagnostic hubs without the need for high capital costs^52,53^ (3) There is increasing population acceptance for cancer detection tests that are minimally invasive (e.g. mammogram, cervical smear) when the benefit of early diagnosis is well explained.^53–56^ The European Randomised Study of Screening for Prostate Cancer (ERSPC) prostate cancer screening trial, for example, has demonstrated that men were willing to be recruited to PSA and DRE studies if the benefits were made clear.^57^ Qualitative studies have further supported that men are willing to undergo screening tests including DRE if supported by information on health care benefits^58^ (4) Increasing acceptance of PSAd as a valuable adjunct in prostate cancer management as mentioned above. (5) The added value from prostate volumes to aid management of male lower urinary tract symptoms (LUTS) in the community. LUTS is itself a growing and large health cost burden to the National Health Service (NHS) and other health economies.^59^ Thus, PSAd may now be at the ideal juncture to be explored as an initial community-based test within the context of an incremental-tiered detection programme. Here, the most resource-intensive and costly test (like mpMRI and biopsy) could be reserved for the most at-risk patients.^48,60^ An alternative to PSAd is the aforementioned blood-based biomarkers that also correct for benign prostate components. These may be easier to administer though comparative performance and costeffectiveness will need to be carefully evaluated.^20,61^

Our study does have important limitations. We used cohorts already referred to secondary care, hence, we cannot make claims about the generalisability of our results to an untested population. PPV and NPV may be altered in a screening context when prevalence for the disease is lower.^62^ However, this would also be the case with PSA hence the relative gain of PSAd over PSA is likely to be similar. Men in our cohort would also have been subject to different local PSA referral thresholds, though we have tried to mitigate this by combining data from three regions in our primary dataset and our validation included men from five different regions. We did not have any central review of imaging or biopsy and, hence, our data represent real-world practice. We also did not attempt to stratify the cohort by MRI positivity or score as our aim was not to re-explore MRI utility as a rule-out or rule-in test. In addition, we also did not include men who had an MRI, but did not proceed to a biopsy as we would not know the true rate of positive or missed cancers in these men. We therefore acknowledge that this means our analysis was limited to those men who had all secondary care tests. Finally, our prostate volume was derived from MRI volumes and not ultrasound which we have advocated above for community use. However, historical and contemporary studies have shown that transrectal ultrasound and MRI are generally concordant for prostate volume estimation.^63–65^

In conclusion, we find that PSA thresholds and PSA age references continue to have similar and modest performance characteristics as reflex tests in modern image-based biopsy pathways. We re-affirm PSAd as a better and more equitable metric to detect csPCa and our data support a reassessment of how men are evaluated to avoid unnecessary secondary care investigations. Combining PSAd with genetic or other factors might further provide personalised risk-stratified-tiered screening models without the need for significant national capital infrastructure investment. This is a particularly important given secondary care costs will only rise in future as prostate cancer is a rapidly growing demographic burden with in an aging male population.

## Supplementary Material

Supplementary Table S1

Supplementary Table S2

## Figures and Tables

**Table 1:** Characteristic of the primary cohort showing median age, PSA, prostate volume, PSAd and cancer detection rates.

Variable (*n* = 2767)	Median (range)
Median age (years)	66.4 (36–90)
PSA (ng/mL)	7.27 (0.1–4751)
Prostate volume (mL)	59.0 (10–225)
PSAd (ng/mL^2^)	0.11 (0.003–148)
Any cancer	1693 (61.1%)
⩾*GG2 cancer*	1071 (38.7%)

PSA: prostate-specific antigen; PSAd: PSA density; GG2: Grade Group 2.

**Table 2:** Detection of ⩾ GG2 disease based on a range of PSA single thresholds for different age groups.

	PSA cut-off	Sensitivity (%)	Specificity (%)	NPV (%)	PPV (%)
All ages (years)	⩾3.0	97.0	8.9	84.0	40.3
	⩾4.0	94.6	14.8	81.2	41.2
	⩾5.0	89.1	27.0	79.6	43.5
50–59 years	⩾3.0	99.1	10.7	97.5	24.8
	⩾4.0	88.0	22.9	86.5	25.3
	⩾5.0	68.5	42.4	81.9	26.1
60–69 years	⩾3.0	97.5	6.6	84.8	32.7
	⩾4.0	95.2	10.4	82.2	33.1
	⩾4.5	92.4	15.4	81.3	33.7
	⩾5.0	87.3	23.2	79.7	34.6
	⩾5.5	79.6	33.6	78.0	35.9
70–79 years	⩾3.0	96.9	9.8	71.9	56.8
	⩾3.5	96.5	11.2	72.3	57.1
	⩾4.0	95.7	12.2	69.9	57.2
	⩾5.0	94.5	16.5	71.1	58.1

NPV: negative predictive value; PPV: positive predictive value; PSA: prostate-specific antigen; PSA in ng/mL.

**Table 3:** Detection of ⩾ GG2disease based on different PSA models across the whole primary cohort (*n* = 2767).

	Sensitivity (%)	Specificity (%)	NPV (%)	PPV (%)
PSA age-reference Model 1*	97.4	8.6	83.8	40.2
PSA age-reference Model 2**	97.4	8.6	83.8	40.2
PSAd ⩾ 0.10 for all	88.4	41.8	85.5	48.2
PSAd ⩾ 0.15 for all	71.0	70.3	79.9	59.4

NPV: negative predictive value; PPV: positive predictive value; PSA: prostate-specific antigen; PSAd: PSA density. PSAd in ng/mL^2^.

* Model 1: PSA⩾2.5, if age⩾49; PSA⩾3.0, if age 50-69; PSA⩾5.0, if age⩾70.

** Model 2: PSA⩾2.5, if age⩾49; PSA>3.0, if age⩾50.

**Table 4:** Relationship between age and MRI-defined prostate volume and comparison of differences between age groups (*n* = 2767).

A			
Age group (years)	Mean volume (mL)	Median volume (mL)	Range
<50	34.3	30.0	17.8–70.0
50–59	50.0	45.0	14.0–200.0
60–69	60.1	52.7	10.3–232.0
70–79	63.3	55.0	2.8–302.0
B			
Age comparisons	Mean difference	*p*-value	CI
50–59 vs ⩽49	15.7	0.008	3.1–28.2
60–69 vs 50–59	10.2	<0.0001	5.4–15.0
70–79 vs 60–69	3.1	0.184	-0.9 to 7.1

CI: confidence interval.

**Table 5:** AUC for PSA and PSAd for predicting any cancer and significant cancer(⩾ GG2 disease).

Disease	PSA		PSAd		p-value
	AUC	95% CI	AUC	95% CI	
Any cancer	0.60	0.58−0.63	0.71	0.69−0.73	<0.0001
⩾GG2	0.68	0.66−0.70	0.78	0.76−0.80	<0.0001

PSA: prostate-specific antigen; PSA density: PSAd; AUC: area under the curve; CI: confidence interval; GG2: Grade Group 2. PSA in ng/mL; PSAd in ng/mL^2^.

**Table 6:** Detection of ⩾ GG2 disease based on a range of PSAd single thresholds for different age groups.

	PSAd cut-off	Sensitivity (%)	Specificity (%)	NPV (%)	PPV (%)
All ages (years)	⩾0.05	99.2	8.7	94.5	39.9
	⩾0.10	88.4	41.8	85.5	48.2
	⩾0.15	71.0	70.3	79.9	59.4
50−59	⩾0.05	100.0	9.8	100.0	23.8
	⩾0.10	86.5	41.8	91.7	29.5
	⩾0.15	66.3	71.5	88.0	39.6
60-69	⩾0.05	99.4	7.9	96.6	32.6
	⩾0.10	91.0	42.5	91.0	41.5
	⩾0.15	72.4	71.4	85.2	53.2
70-79	⩾0.05	98.8	9.2	85.3	58.3
	⩾0.10	87.5	41.6	72.1	65.9
	⩾0.15	71.6	68.0	64.8	73.9

NPV: negative predictive value; PPV: positive predictive value; PSA density: PSAd. PSAd density in ng/mL^2^.
