# Supplementary material for: Re-evaluating the diagnostic efficacy of PSA as a referral test to detect clinically significant prostate cancer in contemporary MRI-based image-guided biopsy pathways
Source: J Clin Urol. Author manuscript; Available in PMC 2023 Aug 23. (PMC7614972; doi:10.1177/20514158211059057)
Supplement: Supplementary Table S1 [file EMS185042-supplement-Supplementary_Table_S1_.docx]

**Supplementary Table S1 – Characteristic of the validation cohort showing median age, PSA, prostate volume and PSA density and cancer detection rates. CPG- Cambridge Prognostic Group**

| **Variable** (*n*=541) | **Median (range)** |
| --- | --- |
| **Median Age (years)** | 66y (43-86) |
| **PSA (ng/ml)** | 8 (0.38-257.8) |
| **Prostate volume (ml)** | 43 (8-313mls) |
| **PSA density (ng/ml^2^)** | 0.18 (0.01-7.10) |
| **Any cancer**  ***≥Grade Group 2 cancer***  ***≥CPG2***  ***≥CPG3*** | 349 (64.5%)  255 (47.1%)  285 (52.6%)  199 (36.7%) |
