# Supplementary material for: Re-evaluating the diagnostic efficacy of PSA as a referral test to detect clinically significant prostate cancer in contemporary MRI-based image-guided biopsy pathways
Source: J Clin Urol. Author manuscript; Available in PMC 2023 Aug 23. (PMC7614972; doi:10.1177/20514158211059057)
Supplement: Supplementary Table S2 [file EMS185042-supplement-Supplementary_Table_S2_.docx]

**Supplementary Table S2 - Detection of** ≥**Grade Group 2 disease,** ≥ **Cambridge Prognostic Group 2 (CPG2) and** ≥ **Cambridge Prognostics Group 3 (CPG3) or more based on different PSA models in the validation cohort (n=539). (NPV- negative predictive value, PPV- positive predictive value). PSA ng/ml, PSA density (PSAd) (ng/mL^2^)**

|  | **Detection of** ≥**Grade Group 2** | | | | **Detection of** ≥**CPG2** | | | | **Detection of** ≥**CPG3** | | | |
| --- | --- | --- | --- | --- | --- | --- | --- | --- | --- | --- | --- | --- |
|  | **Sensitivity** | **Specificity** | **NPV** | **PPV** | **Sensitivity** | **Specificity** | **NPV** | **PPV** | **Sensitivity** | **Specificity** | **NPV** | **PPV** |
| **PSA age reference Model 1*** | 97.6% | 9.09% | 81.3% | 48.9% | 97.5% | 9.7% | 78.1% | 54.6% | 98.0% | 8.2% | 87.5% | 38.3% |
| **PSA age reference Model 2**** | 97.6% | 9.09% | 81.3% | 48.9% | 97.5% | 9.7% | 78.1% | 54.6% | 98.0% | 8.2% | 87.5% | 38.3% |
| **PSAd ≥0.10**  **for all** | 92.5% | 30.8% | 82.2% | 54.4% | 93.0% | 34.0% | 81.3% | 61.1% | 96.9% | 26.6% | 93.5% | 44.2% |

*Model 1: PSA ≥2.5 if age ≤49, PSA ≥3.0 if age 50-69, PSA ≥5.0 if age ≥70

**Model 2: PSA ≥2.5 if age ≤49, PSA ≥3.0 if age ≥50.
